# Supplementary material for: Pediatric post-discharge mortality in resource-poor countries: A protocol for an updated systematic review and meta-analysis
Source: PLoS One. 2023 Feb 24;18(2):e0281732. doi: 10.1371/journal.pone.0281732 (PMC9955921; doi:10.1371/journal.pone.0281732)
Supplement: S1 Table — (DOCX) [file pone.0281732.s002.docx]

**S1 Table. Search Strategy Ovid MEDLINE.**

| **No.** | **Search Category** | **Terms** | **Hits** |
| --- | --- | --- | --- |
| 1 | Post-Discharge Mortality | exp hospitalization/ or (hospital*).ti,ab. |  |
| 2 |  | exp mortality/ or (mortality or death* or fatal* or survival).ti,ab. |  |
| 3 |  | exp follow-up studies/ or exp longitudinal studies/ or Outcome Assessment, Health Care/ or (follow-up* or long term* or postdischarge or (post adj3 discharge) or after discharge or after hospital* or post hospital*).ti,ab. |  |
| 4 |  | 1 and 2 and 3 |  |
| 5 | Low and Low-Middle SDI ​​Countries Names and General Developing Country Terms | (afghanistan or benin or burkina faso or burkina fasso or burundi or urundi or central african republic or chad or cote d’ivoire or cote d’ ivoire or cote divoire or cote d ivoire or ivory coast or democratic republic of the congo or democratic republic congo or eritrea or ethiopia or guinea or guinea bissau or haiti or liberia or madagascar or malagasy republic or malawi or nyasaland or mali or mozambique or portuguese east africa or nepal or niger or pakistan or papua new guinea or new guinea or rwanda or ruanda or senegal or sierra leone or solomon islands or solomon or somalia or south sudan or tanzania or the gambia or togo or togolese republic or uganda or yemen).ti,ab,sh,kf. |  |
| 6 |  | (angola or bangladesh or belize or bhutan or bolivia or cambodia or cameroon or cameron or cameroun or cape verde or cabo verde or comoros or comoro islands or iles comores or congo or djibouti or dominican republic or el salvador or eswatini or swaziland or ghana or gold coast or guatemala or honduras or india or kenya* or kiribati or kyrgyzstan or kirghizia or kirgizstan or kyrgyz republic or kirghiz or laos or lao pdr or "lao people's democratic republic" or lesotho or basutoland or maldives or micronesia or federated states of micronesia or marshall islands or mauritania or mongolia or morocco or myanmar or burma or nicaragua or nigeria or north korea or "democratic people’s republic of korea" or republic of korea or palestine or "sao tome and principe" or sudan or tajikistan or tadjikistan or tadzhikistan or tadzhik or timor leste or east timor or tuvalu or vanuatu or new hebrides or venezuela or zambia or zimbabwe).ti,ab,sh,kf. |  |
| 7 |  | (developing countr* or developing nation? or developing population? or developing world or less developed countr* or less developed nation? or less developed population? or less developed world or lesser developed countr* or lesser developed nation? or lesser developed population? or lesser developed world or under developed countr* or under developed nation? or under developed population? or under developed world or underdeveloped countr* or underdeveloped nation? or underdeveloped population? or underdeveloped world or low income countr* or low income nation? or low income population? or lower income countr* or lower income nation? or lower income population? or underserved countr* or underserved nation? or underserved population? or underserved world or under served countr* or under served nation? or under served population? or under served world or deprived countr* or deprived nation? or deprived population? or deprived world or poor countr* or poor nation? or poor population? or poor world or poorer countr* or poorer nation? or poorer population? or poorer world or developing econom* or less developed econom* or lesser developed econom* or under developed econom* or underdeveloped econom* or low income econom* or lower income econom* or low gdp or low gnp or low gross domestic or low gross national or lower gdp or lower gnp or lower gross domestic or lower gross national or third world or lami countr* or LMIC or sub-saharan africa).ti,ab,sh,kf. |  |
| 8 |  | 5 or 6 or 7 |  |
| 9 | Combining Categories | 4 and 8 |  |
| 10 | Exclusions | (address or autobiography or bibliography or biography or case reports or classical article or clinical conference or comment or congress or consensus development conference or consensus development conference, nih or dataset or dictionary or directory or editorial or "expression of concern" or festschrift or government publication or guideline or historical article or interactive tutorial or interview or lecture or legal case or legislation or letter or meta analysis or news or newspaper article or patient education handout or periodical index or personal narrative or portrait or practice guideline or "review" or "scientific integrity review" or "systematic review" or technical report or video-audio media or webcast).pt. |  |
| 11 |  | (exp animal/ or exp invertebrate/ or animal experiment/ or animal model/ or exp plant/ or exp fungus/) not exp human/ |  |
| 12 |  | exp Neoplasms/ |  |
| 13 |  | 9 not (10 or 11 or 12) |  |
| 14 |  | exp adolescent/ or exp child/ or exp infant/ or (infant disease* or childhood disease*).ti,ab,kf. or (adolescen* or babies or baby or boy? or boyfriend or boyhood or child* or girl? or infant* or juvenil* or kid? or minors or minors* or neonat* or neonat* or newborn* or new-born* or paediatric* or peadiatric* or pediatric* or perinat* or preschool* or puber* or pubescen* or school* or teen* or toddler? or underage? or under-age? or youth*).ti,ab,kf. |  |
| 15 |  | 13 and 14 |  |
| 16 | Limiting | limit 15 to yr="2017-Current" |  |
