# Supplementary material for: Pediatric post-discharge mortality in resource-poor countries: A protocol for an updated systematic review and meta-analysis
Source: PLoS One. 2023 Feb 24;18(2):e0281732. doi: 10.1371/journal.pone.0281732 (PMC9955921; doi:10.1371/journal.pone.0281732)
Supplement: S2 Table — (DOCX) [file pone.0281732.s003.docx]

**S2 Table. Search Strategy Ovid EMBASE.**

| **No.** | **Search Category** | **Terms** | **Hits** |
| --- | --- | --- | --- |
| 1 | Post-Discharge Mortality | exp hospitalization/ or exp hospital discharge/ or (hospital*).ti,ab. |  |
| 2 |  | exp mortality/ or exp mortality risk/ or exp mortality rate/ or (mortality or death* or fatal* or survival).ti,ab. |  |
| 3 |  | exp follow-up/ or exp longitudinal study/ or (follow-up* or long term* or postdischarge or (post adj3 discharge) or after discharge or after hospital*).ti,ab. |  |
| 4 |  | 1 and 2 and 3 |  |
| 5 | Low and Low-Middle SDI Countries Names and General Developing Country Terms | **(afghanistan** or **benin** or **burkina faso** or burkina fasso or **burundi** or urundi or **central african republic** or **chad** or **cote d’ivoire** or cote d’ ivoire or cote divoire or cote d ivoire or ivory coast or democratic republic of the congo or **democratic republic congo** or **eritrea** or **ethiopia** or **guinea** or **guinea bissau** or **haiti** or **liberia** or **madagascar** or malagasy republic or **malawi** or nyasaland or **mali** or **mozambique** or portuguese east africa or **nepal** or **niger** or **pakistan** or **papua new guinea** or new guinea or **rwanda** or ruanda or **senegal** or **sierra leone** or **solomon islands** or solomon or **somalia** or **south sudan** or **tanzania** or **the gambia** or **togo** or togolese republic or **uganda** or **yemen)**.ti,ab,sh,kf. |  |
| 6 |  | **(angola** or **bangladesh** or **belize** or **bhutan** or **bolivia** or c**ambodia** or **cameroon** or cameron or cameroun or **cape verde**  or cabo verde or **comoros** or comoro islands or iles comores or **congo** or **djibouti** or **dominican republic** or **el salvador** or eswatini or **swaziland** or **ghana** or gold coast or **guatemala** or **honduras** or **india** or **kenya*** or **kiribati** or **kyrgyzstan** or kirghizia or kirgizstan or kyrgyz republic or kirghiz or **laos** or lao pdr or "lao people's democratic republic" or **lesotho** or basutoland or **maldives** or micronesia or **federated states of micronesia** or **marshall islands** or **mauritania** or **mongolia** or **morocco** or **myanmar** or burma or **nicaragua** or **nigeria** or **north korea** or "democratic people’s republic of korea" or republic of korea or **palestine** or **"sao tome and principe"** or **sudan** or **tajikistan** or tadjikistan **or** tadzhikistan or tadzhik or **timor leste** or east timor or **tuvalu** or **vanuatu** or new hebrides or **venezuela** or **zambia** or **zimbabwe)**.ti,ab,sh,kf. |  |
| 7 |  | (developing countr* or developing nation? or developing population? or developing world or less developed countr* or less developed nation? or less developed population? or less developed world or lesser developed countr* or lesser developed nation? or lesser developed population? or lesser developed world or under developed countr* or under developed nation? or under developed population? or under developed world or underdeveloped countr* or underdeveloped nation? or underdeveloped population? or underdeveloped world or low income countr* or low income nation? or low income population? or lower income countr* or lower income nation? or lower income population? or underserved countr* or underserved nation? or underserved population? or underserved world or under served countr* or under served nation? or under served population? or under served world or deprived countr* or deprived nation? or deprived population? or deprived world or poor countr* or poor nation? or poor population? or poor world or poorer countr* or poorer nation? or poorer population? or poorer world or developing econom* or less developed econom* or lesser developed econom* or under developed econom* or underdeveloped econom* or low income econom* or lower income econom* or low gdp or low gnp or low gross domestic or low gross national or lower gdp or lower gnp or lower gross domestic or lower gross national or third world or lami countr*).ti,ab,sh,kf. |  |
| 8 |  | 5 or 6 or 7 |  |
| 9 | Combining Categories | 4 and 8 |  |
| 10 | Exclusions | (books or chapter or conference abstract or "conference review" or editorial or erratum or letter or note or "review" or short survey or tombstone).pt. |  |
| 11 |  | (exp animal/ or exp invertebrate/ or nonhuman/ or animal experiment/ or animal tissue/ or animal model/ or exp plant/ or exp fungus/) not (exp human/ or human tissue/) |  |
| 12 |  | exp Neoplasms/ |  |
| 13 |  | 9 not (10 or 11 or 12) |  |
| 14 | Limiting | exp adolescence/ or exp adolescent/ or exp child/ or exp childhood disease/ or exp infant disease/ or (adolescen* or babies or baby or boy? or boyfriend or boyhood or child or child* or child*3 or children* or girl? or infant* or juvenil* or juvenile* or kid? or minors or minors* or neonat* or neonat* or newborn* or new-born* or paediatric* or peadiatric* or pediatric* or perinat* or preschool* or puber* or pubescen* or school or school child* or school* or schoolchild* or schoolchild* or teen* or toddler? or underage? or under-age? or youth*).ti,ab,kf,hw. |  |
| 15 |  | 13 and 14 |  |
| 16 |  | limit 15 to yr="2017-Current" |  |
