# Supplementary material for: Pediatric post-discharge mortality in resource-poor countries: A protocol for an updated systematic review and meta-analysis
Source: PLoS One. 2023 Feb 24;18(2):e0281732. doi: 10.1371/journal.pone.0281732 (PMC9955921; doi:10.1371/journal.pone.0281732)
Supplement: S4 Table — (DOCX) [file pone.0281732.s005.docx]

**S4 Table. Country Classification.**

| **Countries Classified as Low-HDI (2011)**[1] | **Countries Classified as Low-HDI (2016)** [2] | **Countries Classified as Low and Low-Middle SDI (2019)** [3] |
| --- | --- | --- |
| Solomon Islands | Swaziland | Somalia |
| Kenya | Syrian Arab Republic | Niger |
| São Tomé and Príncipe | Angola | Chad |
| Pakistan | Tanzania, United Republic of | Burkina Faso |
| Bangladesh | Nigeria | Mali |
| Timor-Leste | Cameroon | Central African Republic |
| Angola | Papua New Guinea | Burundi |
| Yanmar | Zimbabwe | Mozambique |
| Cameroon | Solomon Islands | Guinea |
| Madagascar | Mauritania | Afghanistan |
| United Republic of Tanzania | Madagascar | Ethiopia |
| Papua New Guinea | Rwanda | Sierra Leone |
| Yemen | Comoros | Benin |
| Senegal | Lesotho | Guinea-Bissau |
| Nigeria | Senegal | South Sudan |
| Nepal | Haiti | Liberia |
| Haiti | Uganda | Democratic Republic of the Congo |
| Mauritania | Sudan | Malawi |
| Lesotho | Togo | Senegal |
| Uganda | Benin | Papua New Guinea |
| Togo | Yemen | Eritrea |
| Comoros | Afghanistan | Madagascar |
| Zambia | Malawi | The Gambia |
| Djibouti | Côte d’Ivoire | Uganda |
| Rwanda | Djibouti | Solomon Islands |
| Benin | Gambia | Côte d'Ivoire |
| Gambia | Ethiopia | Yemen |
| Sudan | Mali | Togo |
| Côte d'Ivoire | Democratic Republic of the Congo | Nepal |
| Malawi | Liberia | Tanzania |
| Afghanistan | Guinea-Bissau | Rwanda |
| Zimbabwe | Eritrea | Haiti |
| Ethiopia | Sierra Leone | Pakistan |
| Mali | Mozambique | Bhutan |
| Guinea-Bissau | South Sudan | Comoros |
| Eritrea | Guinea | Djibouti |
| Guinea | Burundi | Cambodia |
| Central African Republic | Burkina Faso | Angola |
| Sierra Leone | Chad | Zimbabwe |
| Burkina Faso | Niger | Bangladesh |
| Liberia | Central African Republic | Vanuatu |
| Chad |  | Laos |
| Mozambique |  | Cameroon |
| Burundi |  | Honduras |
| Niger |  | Mauritania |
| Democratic Republic of the Congo |  | São Tomé and PrÍncipe |
|  |  | Zambia |
|  |  | Lesotho |
|  |  | Kenya |
|  |  | Timor-Leste |
|  |  | Sudan |
|  |  | Nigeria |
|  |  | Nicaragua |
|  |  | Myanmar |
|  |  | Cape Verde |
|  |  | Guatemala |
|  |  | Kiribati |
|  |  | Tajikistan |
|  |  | Marshall Islands |
|  |  | Morocco |
|  |  | Ghana |
|  |  | North Korea |
|  |  | Maldives |
|  |  | Bolivia |
|  |  | India |
|  |  | Congo |
|  |  | El Salvador |
|  |  | eSwatini (formerly Swaziland) |
|  |  | Federated States of Micronesia (Micronesia) |
|  |  | Palestine |
|  |  | Tuvalu |
|  |  | Dominican Republic |
|  |  | Kyrgyzstan |
|  |  | Belize |
|  |  | Mongolia |
|  |  | Venezuela |

**References**

1. United Nations Development Programme (UNDP) Human Development Report 2011 Team. Human development report 2011: Sustainability and equity: a better future for all [Internet]. New York (US): UNDP; 2011. ISBN: 9780230363311. Available from: https://hdr.undp.org/system/files/documents/human-development-report-2011-english.human-development-report-2011-english
2. UNDP Human Development Report 2016 Team. Human development report 2016: Human development for everyone [Internet]. New York (US): UNDP; 2016. ISBN: 978-92-1-126413-5. Available from: https://sustainabledevelopment.un.org/content/documents/25212016_human_development_report.pdf
3. Global Burden of Disease Collaborative Network [dataset on the Internet]. SDI values: 1990-2020 [XLSX]. Seattle (US): University of Washington; 2020 [cited 2022 Jun 28]. Available from: https://ghdx.healthdata.org/sites/default/files/record-attached-files/IHME_GBD_2019_SDI_1990_2019_Y2020M10D15.XLSX
