# Supplementary material for: Pediatric post-discharge mortality in resource-poor countries: A protocol for an updated systematic review and meta-analysis
Source: PLoS One. 2023 Feb 24;18(2):e0281732. doi: 10.1371/journal.pone.0281732 (PMC9955921; doi:10.1371/journal.pone.0281732)
Supplement: S5 Table — (DOCX) [file pone.0281732.s006.docx]

**S5 Table. Extraction Template for Study Characteristics.**

| Covidence ID | Primary Reference | Additional Reference | Study Start Date | Study End Date | Country | WHO Region 1 | WHO Region 2 | WHO Region 3 | WHO Region 4 |
| --- | --- | --- | --- | --- | --- | --- | --- | --- | --- |
|  |  |  |  |  |  |  |  |  |  |
|  |  |  |  |  |  |  |  |  |  |

| Study Design | If other, specify | Facility Type | If other, specify | Specific Population | Population Group | Enrollment/ Baseline | Total Number enrolled | Number of children enrolled | Eligible Age Range: Lower Limit |
| --- | --- | --- | --- | --- | --- | --- | --- | --- | --- |
|  |  |  |  |  |  |  |  |  |  |
|  |  |  |  |  |  |  |  |  |  |

| Lower Age Limit Units | Eligible Age Range: Upper Limit | Upper Age Limit Units | Comments | Age Estimate Type | Age Estimate | Age Estimate: Units | Age Dispersion Type | Age Dispersion | Age Dispersion: Lower Bound |
| --- | --- | --- | --- | --- | --- | --- | --- | --- | --- |
|  |  |  |  |  |  |  |  |  |  |
|  |  |  |  |  |  |  |  |  |  |

| Age Dispersion: Upper Bound | Female sex: Number Analyzed | Observed Female Pediatric Cases | Female Proportion (%) | Follow-Up Methods | Inclusion Criteria | Exclusion Criteria | Post-Discharge Mortality Survival Curve? (Y/N) | Comments |
| --- | --- | --- | --- | --- | --- | --- | --- | --- |
|  |  |  |  |  |  |  |  |  |
|  |  |  |  |  |  |  |  |  |
