# Supplementary material for: Pediatric post-discharge mortality in resource-poor countries: A protocol for an updated systematic review and meta-analysis
Source: PLoS One. 2023 Feb 24;18(2):e0281732. doi: 10.1371/journal.pone.0281732 (PMC9955921; doi:10.1371/journal.pone.0281732)
Supplement: S6 Table — (DOCX) [file pone.0281732.s007.docx]

**S6 Table. Extraction Template for Outcome Characteristics.**

| Covidence ID | Primary Study Reference | Data Source | Group Number for Analysis | Region | Population Group | Exposure | In-patient mortality: Number Analyzed | Number Died In-Hospital | In-Patient Mortality Proportion (%) | Total Number Discharged Alive |
| --- | --- | --- | --- | --- | --- | --- | --- | --- | --- | --- |
|  |  |  |  |  |  |  |  |  |  |  |
|  |  |  |  |  |  |  |  |  |  |  |

| Total Number Enrolled for Follow-up | Number of Follow-Up Timepoints | Total Duration of Follow-Up | Total Duration of Follow-Up: Units | Total Number LTFU at Final Time Point | Proportion LTFU (%) | Post-Discharge Follow-up Timepoint 0 (Admission/Discharge) | Length of Stay: Units | Length of Stay: Estimate Type | Length of Stay: Dispersion Type | Length of Stay: Estimate |
| --- | --- | --- | --- | --- | --- | --- | --- | --- | --- | --- |
|  |  |  |  |  |  |  |  |  |  |  |
|  |  |  |  |  |  |  |  |  |  |  |

| Length of Stay: Dispersion (SD) | Length of Stay: Dispersion Lower Bound | Length of Stay: Dispersion Upper Bound | Time to Death: Estimate Type | Time to Death: Units | Time to Death: Dispersion Type | Time to Death: Estimate | Time to Death: Dispersion | Time to Death: Dispersion Lower Bound | Time to Death: Dispersion Upper Bound | Number Died at Home |
| --- | --- | --- | --- | --- | --- | --- | --- | --- | --- | --- |
|  |  |  |  |  |  |  |  |  |  |  |
|  |  |  |  |  |  |  |  |  |  |  |

| Proportion Died at Home (%) | Number Died in Hospital | Proportion Died at Hospital (%) | # Who Died in Transit | Proportion Died in Transit (%) | Type of Health Seeking (Hospital/CHW/Unspecified, etc.) | Number Children Who Sought Care | Incidence of Care Seeking | Proportion Who Sought Care (%) | Number of Unplanned Discharge Cases | Proportion Unplanned Discharge (%) |
| --- | --- | --- | --- | --- | --- | --- | --- | --- | --- | --- |
|  |  |  |  |  |  |  |  |  |  |  |
|  |  |  |  |  |  |  |  |  |  |  |

| 6-month neurological sequelae reported? (Y/N) | # of children with neurological sequelae at 6 months |
| --- | --- |
|  |  |
|  |  |
