# Supplementary material for: Pediatric post-discharge mortality in resource-poor countries: A protocol for an updated systematic review and meta-analysis
Source: PLoS One. 2023 Feb 24;18(2):e0281732. doi: 10.1371/journal.pone.0281732 (PMC9955921; doi:10.1371/journal.pone.0281732)
Supplement: S7 Table — (DOCX) [file pone.0281732.s008.docx]

**S7 Table. Extraction Template for Post-discharge Mortality Timeline.**

| Covidence ID | Reference | Data Source (if different than the primary reference) | Group Number for Analysis | Region | Population Group | Exposure | Follow-up Timepoint | PD Re-Hospitalization: Number Analyzed | PD Re-Hospitalization: Number (Cumulative) |
| --- | --- | --- | --- | --- | --- | --- | --- | --- | --- |
|  |  |  |  |  |  |  |  |  |  |
|  |  |  |  |  |  |  |  |  |  |

| PD Re-Hospitalization: Proportion (%) | PD Mortality: Number Analyzed | Time Point Unit | PDM Time Point | Standardized Timepoint (in weeks) | Time Point proportion (%) | Number Died PD (cumulative) | Proportion of Total PDM (%) | Proportion of Total Children Enrolled for Follow-Up (%) | Comments |
| --- | --- | --- | --- | --- | --- | --- | --- | --- | --- |
|  |  |  |  |  |  |  |  |  |  |
|  |  |  |  |  |  |  |  |  |  |
