# Supplementary material for: Pediatric post-discharge mortality in resource-poor countries: A protocol for an updated systematic review and meta-analysis
Source: PLoS One. 2023 Feb 24;18(2):e0281732. doi: 10.1371/journal.pone.0281732 (PMC9955921; doi:10.1371/journal.pone.0281732)
Supplement: S8 Table — (DOCX) [file pone.0281732.s009.docx]

**S8 Table. Extraction Template for Post-discharge Mortality Risk Factors.**

| Covidence ID | Primary Reference | Data Source | Risk Factor Upon Admission | Risk Factor Unit (if applicable) | Admission Risk Factor Group | Admission: Estimate Type | Admission: Adjusted | Admission: Estimate | Admission: Dispersion Type | Admission: Dispersion |
| --- | --- | --- | --- | --- | --- | --- | --- | --- | --- | --- |
|  |  |  |  |  |  |  |  |  |  |  |
|  |  |  |  |  |  |  |  |  |  |  |

| Admission: Lower bound | Admission: Upper Bound | Admission: P-value | Admission: Reference | Admission: Comment | Risk Factor Upon Discharge | Risk Factor Unit (if applicable) | Discharge Risk Factor Group | Discharge: Estimate Type | Discharge: Adjusted | Discharge: Estimate |
| --- | --- | --- | --- | --- | --- | --- | --- | --- | --- | --- |
|  |  |  |  |  |  |  |  |  |  |  |
|  |  |  |  |  |  |  |  |  |  |  |

| Discharge: Dispersion Type | Discharge: Dispersion | Discharge: Lower bound | Discharge: Upper bound | Discharge: P-value | Discharge: Comment | Risk Factor for Re-Admission | Risk Factor Unit (if applicable) | Re-Admission Risk Factor Group | Re-Admission: Estimate Type | Re-Admission: Adjusted |
| --- | --- | --- | --- | --- | --- | --- | --- | --- | --- | --- |
|  |  |  |  |  |  |  |  |  |  |  |
|  |  |  |  |  |  |  |  |  |  |  |

| Re-Admission: Estimate | Re-Admission: Dispersion Type | Re-Admission: Dispersion | Re-Admission: Lower bound | Re-Admission: Upper bound | Re-Admission: P-value | Re-Admission: Reference | Re-Admission: Comment |
| --- | --- | --- | --- | --- | --- | --- | --- |
|  |  |  |  |  |  |  |  |
|  |  |  |  |  |  |  |  |
