# Supplementary material for: Pediatric post-discharge mortality in resource-poor countries: A protocol for an updated systematic review and meta-analysis
Source: PLoS One. 2023 Feb 24;18(2):e0281732. doi: 10.1371/journal.pone.0281732 (PMC9955921; doi:10.1371/journal.pone.0281732)
Supplement: S9 Table — (DOCX) [file pone.0281732.s010.docx]

Critical Appraisal Checklist

Reviewer ______________________________________ Date_______________________________

Author_______________________________________ Year_________ Record Number_________

| **Item** | **Low Risk of Bias** | **Moderate Risk of Bias** | **High Risk of Bias/ Not mentioned/ Unclear** |
| --- | --- | --- | --- |
| 1. Was the sample frame appropriate to address the target population? | □ | □ | □ |
| 1. Were study participants sampled in an appropriate way? | □ | □ | □ |
| 1. Were the study subjects and the setting described in detail? | □ | □ | □ |
| 1. Were valid methods used for the identification of the condition and risk factors?   *Were valid methods used for the identification of the condition?* | □ | □ | □ |
| 1. Were the condition and risk factors measured in a standard, reliable way for all participants?   *Was the condition measured in a standard, reliable way for all participants?* | □ | □ | □ |
| 1. Was there appropriate statistical analysis? | □ | □ | □ |
| 1. Was the response rate adequate, and if not, was the low response rate managed appropriately? | □ | □ | □ |

Comments (including reasoning for moderate or high risk of bias scores)

________________________________________________________________________________________________________________________________________________________________________________________________
